# Seismic anisotropy of the D″ layer induced by (001) deformation of post-perovskite

**DOI:** 10.1038/ncomms14669

**Published:** 2017-04-18

**Authors:** Xiang Wu, Jung-Fu Lin, Pamela Kaercher, Zhu Mao, Jin Liu, Hans-Rudolf Wenk, Vitali B. Prakapenka

**Affiliations:** 1 State Key Laboratory of Geological Processes and Mineral Resources, China University of Geosciences, Wuhan 430074, China; 2 Department of Geological Sciences, Jackson School of Geosciences, The University of Texas at Austin, 1 University Station C1100, Austin, Texas 78712, USA; 3 Department of Earth, Ocean and Ecological Sciences, University of Liverpool, Liverpool L69 3GP, UK; 4 School of Earth and Space Sciences, University of Science and Technology of China, Hefei 230026, China; 5 Department of Earth and Planetary Science, University of California, Berkeley, California 94720, USA; 6 Center for Advanced Radiation Sources, The University of Chicago, Chicago, Illinois 60637, USA

## Abstract

Crystallographic preferred orientation (CPO) of post-perovskite (Mg,Fe)SiO_3_ (pPv) has been believed to be one potential source of the seismic anisotropic layer at the bottom of the lower mantle (D″ layer). However, the natural CPO of pPv remains ambiguous in the D″ layer. Here we have carried out the deformation experiments of pPv-(Mg_0.75_,Fe_0.25_)SiO_3_ using synchrotron radial X-ray diffraction in a membrane-driven laser-heated diamond anvil cell from 135 GPa and 2,500 K to 154 GPa and 3,000 K. Our results show that the intrinsic texture of pPv-(Mg_0.75_,Fe_0.25_)SiO_3_ should be (001) at realistic *P*–*T* conditions of the D″ layer, which can produce a shear wave splitting anisotropy of ∼3.7% with *V*_SH_>*V*_SV_. Considering the combined effect of both pPv and ferropericlase, we suggest that 50% or less of deformation is sufficient to explain the origin of the shear wave anisotropy observed seismically in the D″ layer beneath the circum-Pacific rim.

## Abstract (web summary)

The source of the anisotropic layer (D'' layer) at the bottom of the lower mantle remains unclear. Here, using high pressure and temperature experiments, the authors find that seismic anisotropy observed at the D'' layer is caused by 50% deformation of the minerals post-perovskite and ferropericlase.

Seismological observations have found an anisotropic layer at the bottom of the lower mantle^1^,^2^,^3^, called the D″ layer. The source of shear wave anisotropy in the D″ layer is uncertain, but geophysical models predict large strains along boundaries in the lower mantle. It has been suggested that lower mantle mineral (Mg,Fe)SiO_3_ post-perovskite (pPv), which is believed to be stable at relevant *P*–*T* conditions of the lowermost mantle^4^,^5^, may accommodate the large strains by dislocation slip, resulting in crystallographic preferred orientations (CPOs)^6^,^7^. The existence of CPO may help explain anisotropic regions in the D″ layer^6^,^7^,^8^, such as those beneath the circum-Pacific rim where a series of seismic studies have shown that shear wave radial anisotropy is 1–3% with horizontally polarized shear waves (*V*_SH_) travelling faster than vertically polarized shear waves (*V*_SV_)^8^,^9^,^10^,^11^. Knowledge of the CPO and elasticity of the pPv phase at relevant *P*–*T* and compositional conditions of the lowermost mantle is therefore critical for deciphering enigmatic seismic features of the region, which in turn can help our understanding of the geodynamic processes throughout the history of the deep Earth.

The CPO of pPv has been previously investigated both experimentally and theoretically, but many questions remain (Supplementary Table 1). Radial X-ray diffraction (r-XRD) results for Mg_0.9_Fe_0.1_SiO_3_ pPv (pPv10) synthesized from enstatite and deformed to 157 GPa at room temperature show a (100) texture, which is attributed to slip on the (100) and {110} planes^6^. A subsequent study of MgSiO_3_ pPv, synthesized from vitreous MgSiO_3_, over an extended pressure range from 148 to 185 GPa shows (001) textures, which is attributed to slip on (001)[100] and (001)[010]^7^. It has been suggested that the (100) texture results from transformation of bridgmanite (bgm), while the (001) texture results from stress-induced slip^7^. These different results raise the question as to whether the experimentally observed textures are a result of the bgm-to-pPv phase transformation, or are stress-induced^12^,^13^. Theoretical simulations of MgSiO_3_ pPv at high pressures and 0 K demonstrate a diversity of possible slip systems, including (010), {110} and (001)^14^,^15^,^16^,^17^. On the other hand, elastic anisotropy of D″ layer predicted from global models of mantle flow indicates that the most probable slip of MgSiO_3_ pPv is on the (010) or (001) plane^18^,^19^. MgSiO_3_ pPv is stable only at high pressures (>125 GPa) and high temperatures (>2,000 K) that are challenging for experimental CPO studies. Thus, analogue pPv minerals that are stable at ambient conditions or relatively low *P*–*T* conditions, such as CaIrO_3_, MgGeO_3_, MnGeO_3_ and NaNiF_3_, have been investigated to aid our understanding of the CPO in pPv-structured minerals for interpreting seismic anisotropy of the D″ layer^12^,^13^,^20^,^21^. However, these analogue minerals may not have the same slip systems and indeed do not show systematic CPO patterns in the pPv structure, and thus may not be suitable for understanding the CPO of MgSiO_3_ pPv at lowermost mantle conditions.

Besides disagreement among existing studies, there is also a lack of information for CPO development in Fe-bearing pPv at the lowermost mantle conditions. pPv in the D″ layer is expected to contain iron, where both temperature and iron content affect dislocation slip and texture evolution of materials at high pressure^22^. For example, deformation studies of lower mantle mineral phase ferropericlase (fp) (Mg,Fe)O at high pressures indicate that the {110}<1
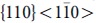
0> slip system dominates at room temperature, whereas {100}<110> becomes equally active at high temperatures^23^,^24^,^25^. Slip on {100}<110> is also more active in (Mg_0.1_,Fe_0.9_)O at room temperature, producing a weaker texture compared with MgO, and therefore demonstrating that iron content affects CPO as well^25^.

Here we have investigated the deformation of Fe-bearing pPv (Mg_0.75_Fe_0.25_SiO_3_; pPv25) using synchrotron r-XRD in a membrane-driven laser-heated diamond anvil cell from 135 GPa and 2,500 K to 154 GPa and 3,000 K. Analysis of the high *P*–*T* diffraction images shows dominant (001) textures, suggesting slip on (001)[100] and (001)[010] or on (001)<110>. Geophysical models of subducting slabs predict that Fe-bearing pPv with slip on (001) would produce a shear wave splitting anisotropy of ∼3.7% with *V*_SH_>*V*_SV_. Our results thus support geodynamic modelling for the mantle flow-induced CPO in pPv that is reflected in seismic observations of the D″ layer beneath the circum-Pacific rim.

## Results

### Texture of Fe-bearing pPv

**Figure 1:**
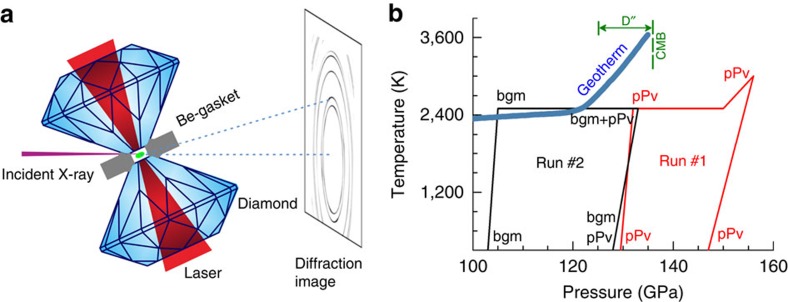
Geometry and *P*–*T* pathway of high-pressure synchrotron r-XRD. (**a**) Schematics of r-XRD in a laser-heated diamond anvil cell; (**b**) experimental *P*–*T* pathway for r-XRD experiments of bgm and pPv (pPv25) plotted with the geotherm of the lowermost mantle^40^.

**Figure 2:**
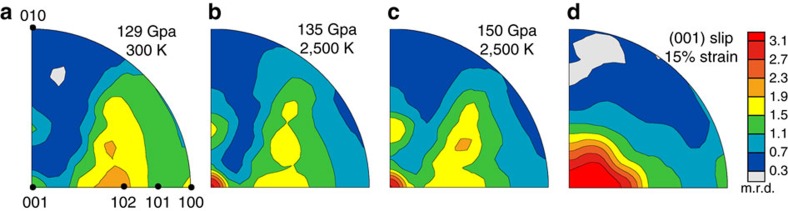
Representative IPFs of pPv at high pressures in run 1. Pole densities are given in multiples of random distribution (m.r.d.) with an m.r.d.=1 representing no preferred orientation. Experimental pPv texture (**a**) at 129 GPa and 300 K right after the phase transformation, (**b**) at 135 GPa and 2,500 K, (**c**) pPv at 150 GPa and 2,500 K. (**d**) Texture predicted with VPSC for slip on (001)[100] and (001)[010] or on (001)<110>. IPFs are shown for compression direction as equal area projections.

Figure 1 demonstrates schematics of synchrotron r-XRD in a laser-heated diamond anvil cell and experimental *P*–*T* pathway in two independent runs (seen Methods for details). Analysis of the r-XRD patterns of the temperature-quenched pPv25 synthesized from enstatite (run 1) at 129 GPa and 300 K (right after the phase transformation) show a crystallographic pole density maximum (texture) near {102} in the inverse pole figures (IPFs) with 2.2 multiples of a random distribution (m.r.d.) (m.r.d.=1 indicates random orientation) and weak secondary maxima at (100) and (001) (Fig. 2a). Upon laser heating of pPv25 to 2,500 K for at least 5 min, the secondary maximum at (001) increased to 3.3 m.r.d (Fig. 2b) and the maximum near {102} became weaker and the secondary maximum at (100) disappeared. The pole densities at (001) and near {102} remained mostly unchanged upon further compression from 135 GPa to 150 GPa at a simultaneous high temperature of 2,500 K. Even at 156 GPa and 3,000 K conditions, there was little change in texture. The larger grain size in these samples, as exhibited from the spotty diffraction patterns, may more readily adopt a deformation texture^13^ with the small increase in differential stress seen in the sample chamber due to thermal expansion during heating. Upon temperature quenching at 156 GPa, the (001) texture became much weaker and the maximum near {102} became dominant again (Supplementary Fig. 1a). Differential stress of the pPv samples at 132 GPa and 2,500 K was relatively low at 0.25 GPa compared with measurements taken at lower temperature^6^,^7^, but increased to 1.50 GPa at 150 GPa (Fig. 2c). In run 2, we were unable to completely transform from bgm to pPv, despite 4 h of laser heating at 133 GPa and 2,500 K. Thus, we analysed textures of both bgm25 and pPv25 during continuous compression and laser heating at 2,500 K. At 133 GPa and 2,500 K, bgm25 displayed no significant texture, whereas pPv25 exhibited a {102} texture maximum (Supplementary Fig. 1), in contrast to the (001) texture maximum seen at 135 GPa and 2,500 K in the run 1. These results suggest that the evolution of texture of pPv25 at high pressures may be sensitive to grain size, differential stress from thermal expansion in the sample chamber, temperature and the degree of phase transformation.

Our observation of a pole density maximum near {102} and a secondary maximum at (100) in pPv25 at 129 GPa, 300 K and differential stress 0.25 GPa following conversion to pPv at 2,000 K for 270 min is similar to that observed for (Mg_0.9_Fe_0.1_)SiO_3_ pPv10 at 145 GPa, 300 K and a differential stress of 7.2–8.5 GPa after conversion to pPv at 1,700–2,000 K for 15–20 min (ref. 6). The (001) texture we observed at 150 GPa, 2500 K and differential stress 1.5 GPa is similar to that of MgSiO_3_ pPv at 148–185 GPa, 300 K and differential stress of 5.3–10.9 GPa after conversion to pPv at 3,500 K for 10 min (ref. 7) (Supplementary Table 1) and to that of analogue MnGeO_3_ pPv at 63–105 GPa, 2,000 K and differential stress 2.4–7.3 GPa (ref. 12). In contrast to previous results, we see a change in texture from {102} at room temperature to (001) upon laser heating, and interestingly this (001) texture is not quenchable from high temperature. Differences in textural evolution between earlier studies and our study may be explained by the differences in (1) temperature and duration of heating during sample synthesis and subsequent deformation (Supplementary Table 1) and (2) use or lack of a pressure medium. Both of these factors affect grain size and differential stress and may slightly influence slip system activity. Most notably, our results taken at simultaneously high pressure and temperature on pPv25 show a much lower differential stress of 0.25–1.5 GPa that is still sufficient to induce deformation by dislocation slip. The much lower differential stress is likely to be due to the combined effects of *in situ* high temperature, a lower experimental pressure range and an NaCl pressure medium.

### Slip systems of Fe-bearing pPv

We have performed viscoplastic self-consistent (VPSC)^26^ polycrystal plasticity simulations to estimate probable slip systems responsible for the observed (001) texture in pPv25. Models are based on previously suggested slip systems listed in Supplementary Table 1. A compressive strain was applied in 1% increments up to 20% strain to 2,000 grains represented as ellipsoids inside of an anisotropic homogenous medium. Summary of relative CRSS and slip system activities for VPSC models are listed in Supplementary Table 2, and IPFs showing texture are shown in Supplementary Fig. 2. We suggest that the texture near {102} is a result of the phase transformation to pPv, whereas the (001) texture is a deformation-induced slip. Thus, we assigned grains an initial preferred orientation similar to our observed {102} transformation texture (Supplementary Fig. 2). The best match to experimental deformation texture (Fig. 2b,c) favours slip on the (001) planes along either the [100] and [010] directions or the <110> direction (Fig. 2d and Supplementary Fig. 2). Thus, slip on (001) should be more representative of the deformation behaviour of pPv in the lowermost mantle.

## Discussion

**Figure 3:**
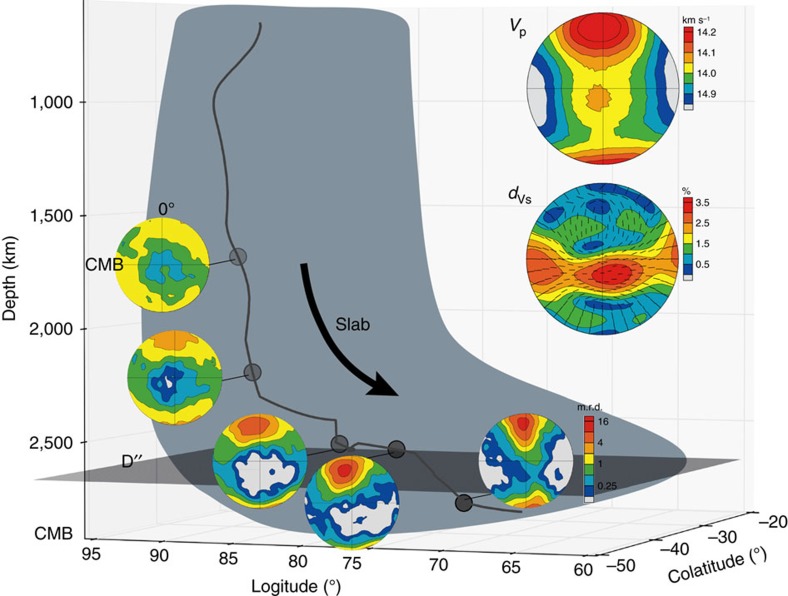
Schematic illustration of possible source of shear wave anisotropy in D″ beneath the circum-Pacific rim. The three-dimensional path of a tracer in the bottom of the slab is shown along with pole figures for pPv at selected depths to show evolution of pPv CPO as the slab descends. The CMB is horizontal and 0° longitude is vertical in all pole figures. The insert are *V*_p_ and *d*_Vs_ of pPv with 100% of deformation accommodated by dislocation slip, where shear wave radial anisotropy (ξ=*V*_SH_^2^/*V*_SV_^2^) is 3.7% and ticks show the orientation of the fast polarized shear wave.

Based on recent geodynamic modelling of the lower mantle, subducting slabs probably sink to the lowermost mantle where the bgm phase is expected to transform to a Fe-bearing pPv phase^4^,^5^,^8^. Seismic velocities resulting from slip on (001)[100] and (001)[010] or on (001)<110> alone in a slab descending to the bottom of the mantle were predicted with a three-dimensional geophysical model^27^, which assumes deformation is accommodated by dislocation slip. Tracers in the model record temperature and strain history as the slab descends. Grain rotations and resulting CPO are calculated from incremental strain recorded by the tracer, relative critical resolved shear stress of slip systems and starting grain orientations. The transformation texture (Fig. 2a) was assigned as the initial texture in the slab. Texture results for one tracer at the bottom of the slab and ∼100 km above the core-mantle boundary (CMB) were combined with single-crystal elastic constants of pPv at 130 GPa and 3,000 K (ref. 28) to calculate *P*- and *S*-wave velocities. The path of the tracer and CPO evolution are shown in Fig. 3. Results show that slip on (001)[100] and (001)[010] or on (001)<110> alone produces a fast horizontal polarization direction with 3.7% shear wave radial anisotropy (the inset of Fig. 3, *d*_Vs_), consistent with seismic observations of *V*_SH_>*V*_SV_ in D″ layer beneath the circum-Pacific rim^7^,^10^,^11^.

Previous investigations of the source of anisotropy in the D″ have shown that the expected texture of bmg in D″ layer does not produce a seismic signature consistent with seismic observations and thus is not expected to contribute significantly to the observed seismic anisotropy in the region^27^. (Mg,Fe)O fp with high *V*_s_ anisotropy of ∼40% and abundance of ∼25% volume in the D″ layer, has been suggested as a source for the observed anisotropy in D″ (ref. 23). Here we also ran the simulation with 75 vol.% pPv and 25 vol.% fp to investigate the effect of (Mg,Fe)O on seismic velocities in D″ and found that the fastest shear wave polarization directions are tilted subparallel to the CMB with a shear wave radial anisotropy of 3.42% with *V*_SH_>*V*_SV_ (Supplementary Fig. 3), which is similar to that of the pPv (Fig. 3). Thus, our results would suggest that pPv is mostly responsible for the seismic signature observed in the D″ with possibly some contribution from (Mg,Fe)O. However, results here do not take into account grain–grain interactions between stronger pPv grains and presumably weaker (Mg,Fe)O grains, as they are deformed together, which has been shown to affect CPO (for example, Kaercher *et al*.^29^).

**Figure 4:**
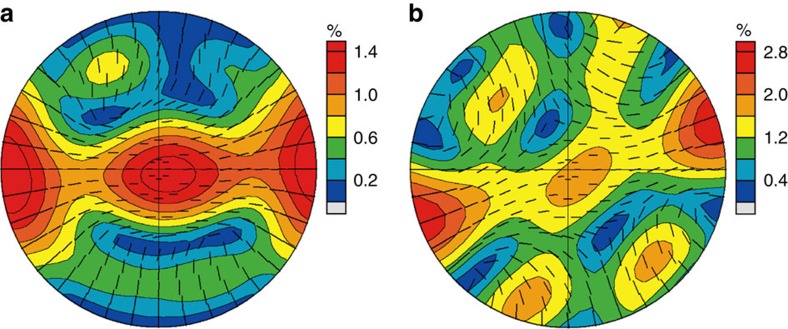
Shear wave splitting maps for pPv and pPv+fp with 50% of deformation accommodated by dislocation slip. (**a**) For pure pPv, shear wave radial anisotropy (*ξ*) is 2.65% and the range of shear wave splitting is 0–1.61%. (**b**) For 75% pPv and 25% (Mg,Fe)O, *ξ* is 2.07% and shear wave splitting is 0.14–2.72%. Ticks show the orientation of the fast polarized shear wave.

Selected regions of the D″ layer beneath the circum-Pacific rim are denoted in the set of Supplementary Fig. 4, where the shear wave splitting anisotropy of Caribbean (1), North West Pacific (3) and Antarctic ocean (5) are about 1–1.5%, and others (2,4) are about 0.5% from the literature after 2000 (Supplementary Table 2). Shear wave splitting anisotropy values for deformation accommodated entirely by dislocation slip (Fig. 3 and Supplementary Fig. 3) are larger than those of geophysics observations, meaning that not all deformation may be accommodated by dislocation slip in these regions. Thus, we also ran a simulation with only 50% of deformation accommodated by dislocation slip for pPv and pPv+fp (Fig. 4), where the shear wave radial anisotropy reduces to 2.65% and 2.07%, respectively, consistent with previous results^30^. This suggests that 50% or less of deformation accommodated by slip is enough to explain the source of shear wave anisotropy in the D″ layer beneath the circum-Pacific rim (Supplementary Fig. 4). Future experimental studies on elastic constants and CPO of FeAl-bearing pPv and fp at relevant *P*–*T* conditions of the D″ layer are necessary to further confirm the origin of the shear wave splitting in the region.

## Methods

### High-pressure synchrotron r-XRD experiments

Polycrystalline (Mg_0.75_,Fe_0.25_)SiO_3_ enstatite was used as the starting material for the synthesis of bgm and pPv at high *P*–*T* conditions^31^. A modified symmetric diamond anvil cell (DAC) with two large side openings of 2*θ*=30° was integrated with a helium-driven membrane unit to apply pressure for *in situ* r-XRD experiments. A pair of beveled diamond anvils with a culet size of 100–300 μm in diameter was used to pre-indent a Be gasket of 3 mm in diameter with an initial central thickness of ∼100 μm to ∼30 μm-thick. Subsequently, a hole of 120 μm was drilled in the pre-indented area and filled with a cubic boron nitride (cBN) gasket insert. This assemblage was further compressed to 25 GPa, compressing the cBN gasket insert to 25 μm thickness, and a hole of 80 μm was drilled in the cBN. The sample was sandwiched between two 5 μm platelets of NaCl (run 1: for synthesis of pPv25 directly from enstatite) or KCl (run 2: for synthesis of pPv25 from bgm following conversion of bgm from enstatite) for thermal insulation and pressure calibration (the pressure uncertainty is estimated to be about 2–4 GPa based on their well-known *P*–*T*–*V* equation of states^32^,^33^) and inserted into the cBN sample chamber. Samples were ∼15 μm thick and 40 μm in diameter.

High *P*–*T* r-XRD experiments were conducted at beamline 13-ID-D (GSECARS) of the Advanced Photon Source (APS) at Argonne National Laboratory. The double-sided laser heating system was modified to accommodate *in situ* collection of r-XRD patterns of bgm and pPv25 at simultaneous high *P*–*T* (ref. 34) (Fig. 1). To avoid contamination of the diffraction peaks from Be gasket, an incident X-ray beam with a monochromatic wavelength of 0.3344 Å was focused down to 3 μm in diameter (full-width at half maximum) onto the sample at an angle of 67.5° from the compression axis of the DAC. To ensure the diffraction images were taken only from the heated area of the sample, X-ray-induced fluorescence from the NaCl or KCl layers was used to align the X-ray spot of ∼3 μm with the laser heating spot of ∼15 μm on both sides of the sample. Temperatures of the heated samples were derived from fitting the grey-body thermal radiation to Planck’s radiation function^34^, where we adjusted the laser power to keep the peak temperatures within ∼50 K on each side. XRD patterns were recorded using a MarCCD detector with an exposure time of 20–30 s. Instrument parameters of the CCD (charge-coupled device) were calibrated with a LaB_6_ standard.

In run 1, the enstatite sample assemblage was slowly compressed to 132 GPa where it became amorphous and then laser-heated to 2,000 K, to synthesize the pPv phase directly. Upon temperature quenching after laser heating, analysis of the XRD patterns confirmed the occurrence of the pPv25 phase without other unknown diffraction peaks at 129 GPa and 300 K. We intentionally kept the heating temperature at 2,500 K for the synthesis relatively low, to avoid potential strong grain, growth-induced spotty diffraction patterns that can affect the analysis of the textures. The pPv25 sample at 129 GPa and 300 K was then laser heated to 2,500 K and eventually compressed to 150 GPa, while maintaining the high temperature. At 150 GPa, the sample was further heated up to 3,000 K where the pressure increased to 156 GPa. In run 2, enstatite was compressed to 103 GPa at 300 K and then laser-heated to 1,500 K, to synthesize bgm crystals. The sample was further heated to 2,500 K and consequently compressed from 105 GPa to 133 GPa in 3–5 GPa intervals, to simulate the transformation of bgm to pPv and to understand how the bgm to pPv transformation affects the textures of the pPv phase at high *P*–*T* (Fig. 1). These experiments confirm the conclusions of dominant (001) slip in pPv (refs 7, 13) and also help explain the relative texture intensity between bmg and pPv at lowermost mantle conditions. Results differ from conclusions of dislocation simulations ref. 35.

### Data analysis and viscoplastic simulations

Experimental r-XRD images were initially processed using the Fit2D^36^ software to mask saturated diffraction spots and produce unrolled diffraction patterns. As the bottom of the tilted DAC blocked some of the diffraction patterns, ∼180° of Debye rings were collected and analysed, which is still sufficient for textural analysis as only 90° is needed. The unrolled patterns were sliced into 36 spectra at every 5° along the azimuthal angle *η* and integrated for each slice. The spectra were then refined using the Rietveld method as implemented in the MAUD software package^37^. We followed the same steps as Wenk *et al*.^37^ to extract available information. Together with theoretical elastic constants of pPv-MgSiO_3_ (ref. 28), the moment pole stress model^38^ was used to calculate elastic lattice strains from sinusoidal variations in peak position. One typical refinement of pPv25 at 150 GPa and 2,500 K in run 1 is shown in Supplementary Fig. 5. Texture is represented in IPFs that were processed in the software BEARTEX^39^. VPSC polycrystal plasticity simulations were performed to estimate probable slip systems responsible for the development of the experimentally observed textures^26^ and are discussed in more detail in the Supplementary Information (Supplementary Fig. 2 and Supplementary Table 2).

### Data availability

The data sets generated and/or analysed during the current study are available from the corresponding authors.

## Additional information

**How to cite this article:** Wu, X. *et al*. Seismic anisotropy of the D″ layer induced by (001) deformation of post-perovskite. *Nat. Commun.* **8,** 14669 doi: 10.1038/ncomms14669 (2017).

**Publisher’s note**: Springer Nature remains neutral with regard to jurisdictional claims in published maps and institutional affiliations.

## Supplementary Material

Supplementary Information Supplementary Figures, Supplementary Tables and Supplementary References
