# Supplementary material for: Seismic anisotropy of the D″ layer induced by (001) deformation of post-perovskite
Source: Nat Commun. 2017 Apr 18;8:14669. doi: 10.1038/ncomms14669 (PMC5501973; doi:10.1038/ncomms14669)
Supplement: Supplementary Information — Supplementary Figures, Supplementary Tables and Supplementary References [file ncomms14669-s1.pdf]

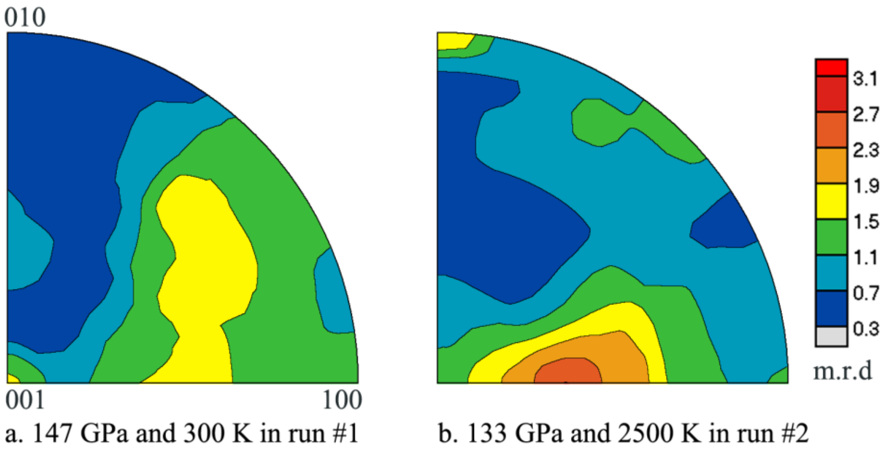

Supplementary Figure 1 | The inverse pole figures of pPv at (a) 147 GPa and 300 K in run#1 (after high-temperature quenched from 3000K) and (b) at 133 GPa and 2500 K in run#2 (coexisted with Bridgmanite).

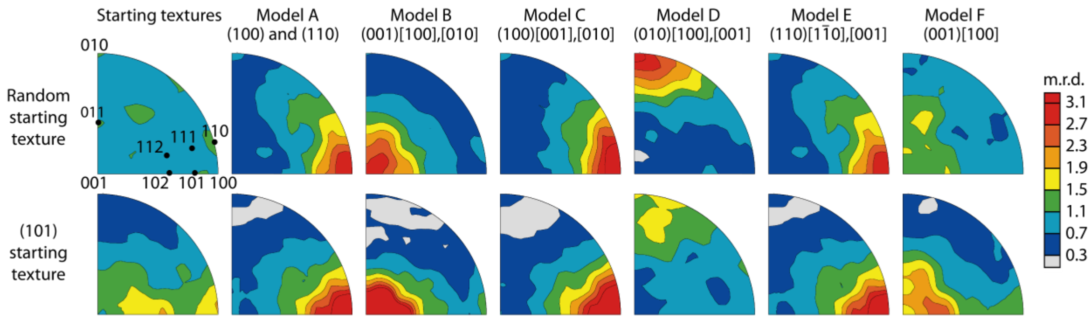

Supplementary Figure 2 | IPFs showing texture produced by VPSC at 20% strain. Models given in Supplementary Table 2 are based on slip systems previously suggested for pPv (Supplementary Table 1). Models were run once with crystals initially randomly oriented (top row) and once with a starting texture similar to the transformation texture seen in experiments (Supplementary Fig. 1). Model B and Model H produce the closest match to experimental results. Pole densities are given in multiples of random distribution (m.r.d.). Major crystallographic directions are labeled in the first IPF. Additional model VPSC textures are shown for isostructural $CaIrO_3$ in reference 9.

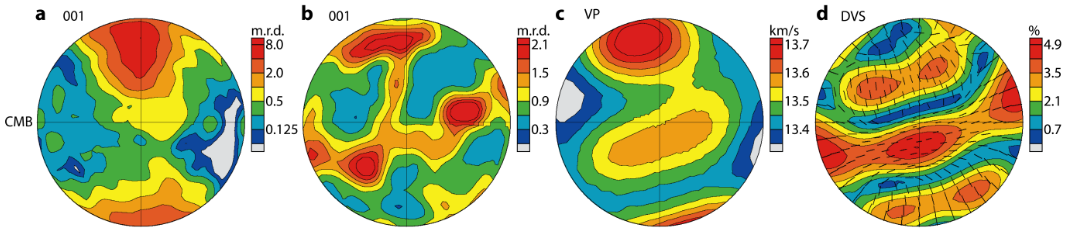

Supplementary Figure 3 | Texture and anisotropy predicted for a slab containing 75 vol.% pPv and 25 vol.% fp with 100% of the strain. The simulations are predicted by the 3D tracer model being accommodated by dislocation slip. Dominant slip systems in pPv are (001)[100] along with either (001)[010] or (001)<110>, and in (Mg,Fe)O, <110>{$\bar{1}$10}. Core mantle boundary (CMB) is horizontal in all plots. (a) pole figure of (001) poles in pPv shows that (001) pPv planes preferrentially align parallel to the CMB. (b) pole figure of (001) poles in MgO showing (001) planes tilted sub-parallel to the CMB. (c) *P*-wave velocity map calculated from pPv texture (a) and (Mg,Fe)O texture (b) and elastic constants of pPv at 130 GPa and 3000 K from the literature[10], and those of (Mg,Fe)O fp with LS of iron from the literature[11]. (d) Shear wave splitting map. Ticks show the orientation of the fast polarized shear wave. Shear wave radial anisotropy (ξ) for 75% pPv and 25% (Mg,Fe)O is 3.42% with $V_{sh}>V_{sv}$, range of shear wave splitting is 0-4.88% (plotted in d).

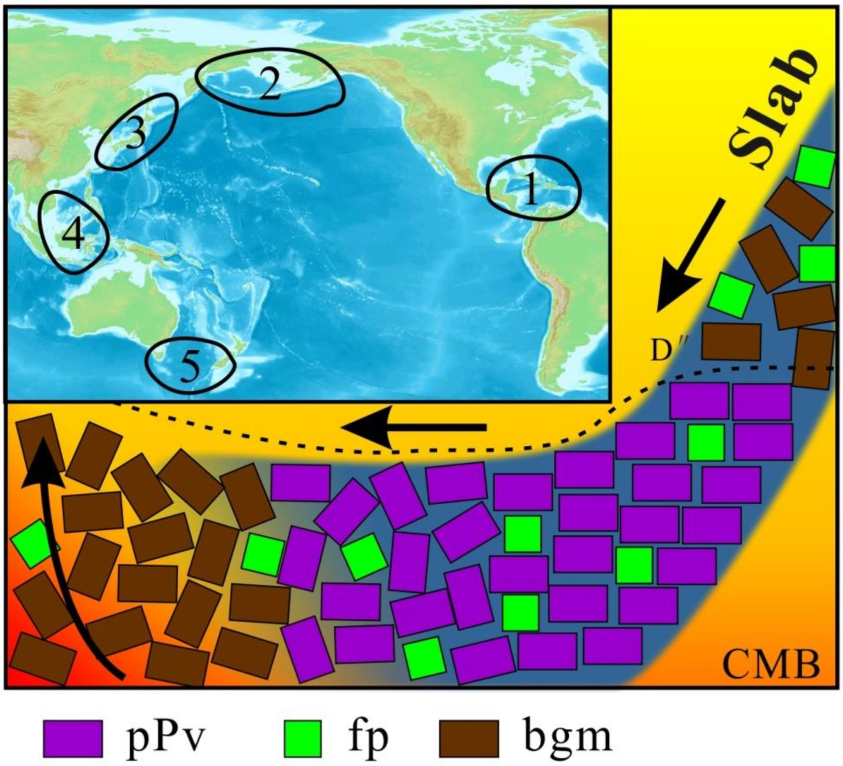

Supplementary Figure 4 | Schematic illustration of possible source of shear wave anisotropy in D′′ beneath the circum-Pacific rim. The subducted slab to D′′ layer undergoes high-strain deformation, resulting in CPO of sub-parallel to CMB. In the hotter regions, pPv converts back to bgm with random orientation[4,12]. The insert shows the selected regions of D′′ layer are with $V_{SH}>V_{SV}$ and their shear wave radial anisotropy δ_{Vs} are 0.8-2.2% for Caribbean region (1) [5,16], 0-0.9% for Alaska region (2) [17], 0.80-1.86% for North West Pacific region (3) [18], 0.5% for Southeast Asia region (4) [19] and 1.0% for Antarctic Ocean region (5) [20].

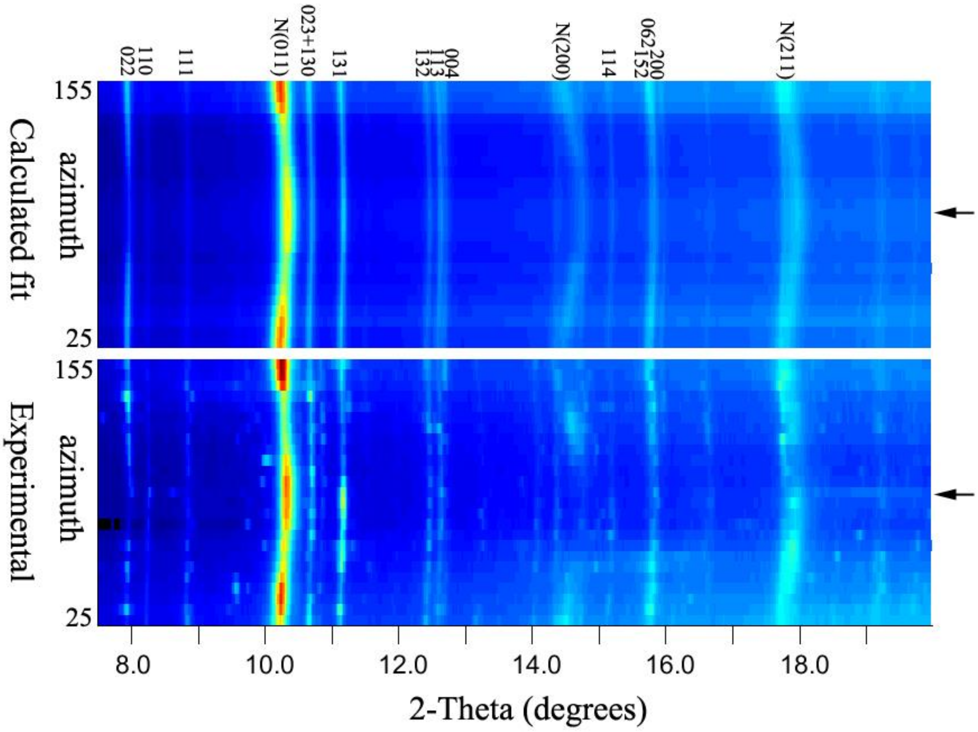

Supplementary Figure 5 | XRD pattern of pPv25 at 150 GPa and 2500K in run #1. The bottom is "Unrolled" diffraction image from 25~155 degree in azimuth. Variations in peak position of pPv are observed to be small, indicating that elastic stresses in the sample chamber are low. Systematic intensity variations of the peak demonstrate the texture distributions. The top is the Rietveld fit, where diffraction peaks of pPv and pressure medium NaCl are denoted by hkl and N(*hkl*), respectively. Black arrows indicate the compression direction.

Supplementary Table 1 | Summary of the slip systems in (Mg,Fe)SiO_3-pPv from experimental and theoretical results

Composition	Synthesis conditions	P-T range	Dominant slip systems	Differential stress (GPa)	References
$(Mg_{0.9},Fe_{0.1})SiO_3$	145GPa and 1700K, 20 min; 2000K, 15 min	145-157 GPa at 300K	(100) and {110}	7.2-8.5	[1]
$MgSiO_3$	148GPa and 3500K, 10 min	148-185 GPa at 300K	(001)[100] and (001)[010]	5.3-10.9	[2]
$(Mg_{0.6}Fe_{0.4})SiO_3$	140 GPa and 2000K, time ?	145-95 GPa at 300 K	{100} or{110}		[3]
$(Mg_{0.75}Fe_{0.25})SiO_3$	130 GPa and ~2000K, 270min	130-156 GPa at 2500-3000 K	(001)[100] and (001)[010] or (001)<110>	0.25-1.5	this study
$MgSiO_3$	Theoretical simulation	0 K	(010)<br>{110}<br>(001)[100]		[4-6]<br>[7]<br>[8]
$(Mg_{0.92}Fe_{0.08})SiO_3$			(001)[100]		[8]

Supplementary Table 2 | Summary of relative CRSS and slip system activities for VPSC models at 20% strain from a {102} starting texture. Models A-F are based on previously suggested slip systems listed in Table 1. Lowering relative CRSS of secondary slip systems (e.g. lowering CRSS for (100) slip systems in Model D to 5), reduces the maximum pole density but does not change the location of the maximum pole densities shown in Supplementary Fig. 2. In these simulations, cell parameters are *a*=2.456Å, *b*=8.042 Å, *c*=6.093 Å[12].

		(001) [100]	(001) [010]	(001) {110}	(100) [001]	(100) [010]	(010) [100]	(010) [001]	{110} <110>	{110} [001]	{111} <101>	Refs.
Model A	CRSS	50	50	--	1	1	50	50	1	1	50	[1]
	Act %	0	0	--	9.9	6.9	0	0	48.8	34.4	0	
Model B	CRSS	1	1	--	50	50	50	50	50	50	50	[2]
	Act %	55.2	41.2	--	0	0.3	0.3	0	0.7	0	2.2	
Model C	CRSS	50	50	--	1	1	50	50	50	50	50	[15]
	Act %	0	0.3	--	55.5	39.2	0	0.3	2.7	0	1.9	
Model D	CRSS	50	50	--	50	50	1	1	50	50	50	[4,7]
	Act %	0.4	0	--	0.4	0	47.5	48.2	0.2	0.6	2.8	[8]
Model E	CRSS	50	50	--	50	50	50	50	1	1	50	[6,13]
	Act %	0	0	--	0	0	0	0	56	44	0	
Model F	CRSS	1	50	--	50	50	50	50	50	50	50	[4]
	Act %	46.1	6.2	--	0	3	3	6.2	9.7	0.3	25.5	
Model G	CRSS	50	1	--	50	50	50	50	50	50	50	NA
	Act %	3.2	35.9	--	3.2	3.0	3.0	0.0	9.6	5.6	36.4	
Model H	CRSS	50	50	1	50	50	50	50	50	50	50	NA
	Act %	0	0	94.7	0	0.4	0.4	0	1.0	0	3.4	

Supplementary References

1. Merkel, S., McNamara, A. K., Kubo, A., Speziale, S., Miyagi, L., Meng, Y., Duffy, T. S., Wenk, H. R. Deformation of (Mg,Fe)SiO_3 post-perovskite and D″ anisotropy. *Science* 316, 1729–1732 (2007).
2. Miyagi, L., Kanitpanyacharoen, W., Kaercher, P., Lee, K. K. M., Wenk, H. R. Slip systems in $MgSiO_3$ postperovskite: Implications for D″ anisotropy. *Science* 329, 1636–1638 (2010).
3. Mao, W. L., Meng, Y., Mao, H. K. Elastic anisotropy of ferromagnesian post-perovskite in Earth's D" layer. *Physics of the Earth and Planetary Interiors* 180, 203-208 (2010).
4. Oganov, A. R., Ono, S. Theoretical and experimental evidence for a post-perovskite phase of $MgSiO_3$ in the Earth's D″ layer. *Nature* 430, 445-448 (2004).
5. Nowacki, A., Wookey, J., Kendall, J. M. Deformation of the lowermost mantle from seismic anisotropy. *Nature* 467, 1091-1096 (2010).
6. Goryaeva, A., Carrez, P., Cordier, P. Modeling defects and plasticity in $MgSiO_3$ postperovskite: Part 2—screw and edge [100] distance locations. *Phys. Chem. Minerals* 42, 793-803 (2015).
7. Oganov, A. R., Martonak, R., Laio, A., Raiteri, P., Parrinello, M. Anisotropy of Earth's D″ layer and stacking faults in the $MgSiO_3$ post-perovskite phase. *Nature* 438, 1142–1144 (2005).
8. Metsue, A., Tsuchiya, T. Shear response of Fe-bearing in $MgSiO_3$ post-perovskite at lower mantle and pressure. *Proc. Jpn. Acad. Ser.* B89, 51-57 (2013).
9. Miyagi, L., Nishiyama, N., Wang, Y., Kubo, A., West, D.V., Cava, R.J., Duffy, T.S., Wenk, H-R. Deformation and texture development in $CaIrO_3$ post-perovskite phase up to 6 GPa and 1300 K. *Earth Planet. Sci. Lett.* 268, 515-525 (2008).
10. Wentzcovitch, R. M., Tsuchiya, T., Tsuchiya, J. $MgSiO_3$ post perovskite at D″ conditions. *Proc. Natl. Acad. Sci.* USA 103, 543-546 (2006).
11. Wu, Z., Justo, J., Wentzcovitch, R. M. Elastic anomalies in a spin-crossover system: ferropericlase at lower mantle conditions. *Phys. Rev. Lett*. 110, 228501 (2013).
12. Murakami, M., Hirose, K., Kawamura, K., Sata, N., Ohishi, Y. Post-perovskite phase transition in $MgSiO_3$. *Science* 304, 855-858 (2004).
13. Yoneda, A., Fukui, H., Xu, F., Nakatsuka, A., Yoshiasa, A., Seto, Y., Ono, K., Tsutsui, S., Uchiyama, H., Baron, A. Q. R. Elastic anisotropy of experimental analogues of perovskite and post-perovskite help to interpret D" diversity. *Nature Commun*. 5, 3453 (2014).
14. Miyagi, L., Kanitpanyacharoen, W., Stackhouse, S., Militzer, B., Wenk, H. R. The enigma of post-perovskite anisotropy: deformation versus transformation texture. *Phys. Chem. Minerals* 38, 665–678 (2011).
15. Tsuchiya, T., Tsuchiya, J., Umemoto, K., Wentzcovitch, R. M. Phase transition in $MgSiO_3$ perovskite in the earth's lower mantle. *Earth Planet. Sci. Lett.* 224, 241–248 (2004).

16. Maupin, V., Garnero, E. J., Lay, T., Fouch, M. J. Azimuthal anisotropy in the D″ layer beneath the Caribbean. *J. Geophys. Res. Solid Earth* 110, B08301 (2005).
17. Fouch, M. J., Fischer, K. M., Wysession, M. Lowermost mantle anisotropy beneath the Pacific: Imaging the source of the Hawaiian plume. *Earth Planet. Sci. Lett*. 190, 167-180 (2001).
18. Wookey, J., Kendall, J. M., Rümpker, G. Lowermost mantle anisotropy beneath the north Pacific from differential S—ScS splitting. *Geophys. J. Inter.* 161, 829-838 (2005).
19. Thomas, C., Wookey, J., Simpson, M. D″ anisotropy beneath southeast Asia. *Geophys. Res. Lett.* 34, L04301 (2007).
20. Usui, Y., Hiramatsu, Y., Furumoto, M., Kanao, M. Evidence of seismic anisotropy and a lower temperature condition in D″ layer beneath Pacific Antarctic Ridge in the Antarctic Ocean. *Phys. Earth Planet. Interiors* 167, 205-216 (2008).
